# The Relationship between Exposure to Pesticides and the Occurrence of Lymphoid Neoplasm

**Published:** 2012-06-30

**Authors:** M Zakerinia, M Namdari, S Amirghofran

**Affiliations:** 1 Department of Internal Medicine, Shiraz University of Medical Sciences, Shiraz, Iran; 2 Hematology Research Center, Shiraz University of Medical Sciences, Shiraz, Iran; 3 Kish International Branch, Shiraz University of Medical Sciences, Shiraz, Iran

**Keywords:** Exposure, Pesticide, Non-Hodgkin lymphoma, Hodgkin lymphoma, Multiple myeloma

## Abstract

**Background:**

The etiology of malignant lymphoma is still largely unknown. This study determines the relationship between exposure to pesticides and the occurrence of lymphoid neoplasms in Shiraz, Southern Iran.

**Methods:**

Between 2007 and 2008, in a case control study conducted in Nemazee Hospital in Shiraz, Southern Iran, 200 subjects diagnosed with lymphoma according to the World Health Organization (WHO) classification were enrolled. Controls (n=200) were frequency matched to the cases by sex, age, and center. Subjects who were a farmer were compared with all other occupations.

**Results:**

Out of the 200 cases that were diagnosed as lymphoid neoplasms, 100 were non-Hodgkin's lymphoma, 54 Hodgkin's lymphoma and 46 multiple myeloma. Seventy two percent of the NHL's were of the B-cell type, 15% of the T-cell type and the rest were not classified. Furthermore, subjects exposed to pesticides were at an increased risk of non-Hodgkin lymphoma and MM, but not Hodgkin lymphoma.

**Conclusion:**

Risk of non-Hodgkin lymphoma and MM was highest for exposure to pesticides, among them, insecticide's risk was confirmed.

## Introduction

In the past decades, the incidence of non-Hodgkin’s lymphoma has doubled in almost all western countries.[1] The causes in the increase of non-Hodgkin’s lymphoma are largely unexplained. A known predisposing factor that is strongly associated with non-Hodgkin’s lymphoma is immunosuppression.[2] A few other risk factors have been identified, including exposure to infectious agents,[3][4] and chemical and agricultural exposures[1][2][4] while many of these exposures were occupational.[4][5] Attention has focused on farmers because they experience several of these exposures, including exposure to zoonosis, pesticides, and other chemicals such as fertilizers and solvents,[5] and particularly those using pesticides, have been identified to be at an increased risk of lymphoid neoplasms. However, these findings have not been confirmed.[6] Studies that investigated the genetic basis of lymphoma showed a relation between farming and lymphoma and pesticide exposure and lymphoma.[4][7] Many pesticides are carcinogenic in animals and can act through several mechanisms to cause cancer in animals, but their role in human carcinogenesis remains partly unclear. Alteration of immune function is a known cause of human cancer and may be responsible for the carcinogenesis associated with some pesticides.[1]

The objective of this study was to describe the risk of lymphoid neoplasms among farmers and other subjects exposed to pesticides, with respect to type of exposures, duration of exposures and evaluate other risk factors such as smoking, family history of cancers, HBV, HCV and HIV infections in a case control study in Nemazee Hospital, Shiraz, Sothern Iran.

## Materials and Methods

Cases were all subjects over 18 years old diagnosed with lymphoma (Hodgkin and non-Hodgkin's lymphoma) and multiple myeloma admitted in Nemazee Hospital between January 2007 and April 2008. The diagnosis was verified by histology and was supplemented by immunohistochemistry tests. Cases were categorized according to the World Health Organization (WHO) classification for Neoplastic Diseases of the Lymphoid Tissues and included B cell, T cell, multiple myeloma, and Hodgkin’s lymphoma. Ineligibility criteria included a history of organ transplantation, severe systemic diseases or a diagnosis of chronic lymphocytic leukemia, or B or T-cell lymphoblastic leukemia.

Of the initial sample, 14 cases were excluded, due to wrong or uncertain diagnosis. Finally, 200 cases were included. Of 200 participating lymphoma patients (participation rate=93.45%), 54 suffered from Hodgkin’s lymphoma, 72 from B-non-Hodgkin’s lymphoma, 15 from T-non-Hodgkin’s lymphoma, 13 from unknown type of Non-Hodgkin’s lymphoma and 46 from multiple myeloma.

Controls were hospital patients randomly chosen among subjects admitted in Nemazee Hospital Emergency Room. Controls were frequency matched by age, sex, state of residence, and vital status at the time of interview. A variety of diagnoses were included. Patients with hematopoietic cancer, systemic infection, previous history of organ transplantation, or patients with severe systemic diseases were excluded. Interviews were obtained from 200 controls. No upper age limit was set for cases and controls, but all subjects had to be able to answer a questionnaire.

Interviews were conducted directly with the subjects or with their next-of-kin if the subjects were in-capacitated (22% of case objects and 20.3%of control objects). Patients were asked to participate in research about the effects of the infections, occupations and environment on the development of lymphoma, and verbal consent was obtained.

Cases and controls were interviewed and answered a questionnaire which contained detailed information on sociodemographic characteristics (including age, sex, place of residence, educational status), familial history (including types of cancer), medical history about presenting disease (including duration of disease, history of previous admission, duration of hospital admission, type of treatment), past medical history and complete occupational history.

Questions regarding occupational history were asked for each job ever held for at least one year. The interviewer listed a series of exposures of interest (for example, pesticides, petroleum, dye,…) for each job. For each of these exposures, the subject specified if the exposure occurred during work, determined the kind of exposure, explained the reason for the exposure, and specified the years of exposure. After finishing the job history, the subject was asked about any extra jobs in crop farming.

In this study, the term pesticide referred primarily to herbicides, insecticides, and fungicides.

Subjects were asked whether they had used or personally handled specific pesticides; for which kind of crops they used pesticide; the year of first use and last use; and the year of use for specific pesticides, the application method used most often; use of protective equipment were recorded. However, duration and intensity measures were obtained for insecticides, herbicides and fungicides, but not for individual pesticides. Non-occupational use of pesticides (home, garden) was included. There were few individuals who were completely free of being exposed to pesticides. The duration of insecticide and herbicide use was categorized into 2 groups (< median and median years of exposure) based on the distribution among subjects who used insecticides and herbicides, respectively. The specific chemicals used were reported in an open-ended question at the end of each section. For assistance with exposure assessment, information on what kinds of pesticides were known to be used for different types of crop and pest type (insect, weed, etc.) in Fars's cities, Jahad Agricultural Support Services in Shiraz assisted with statistics about the distribution of pesticides in Fars in the last 5 years.

At first, we analyzed the relation between occupational exposure and lymphomas as a whole (n=200). Odds ratios (OR) and 95% confidence intervals (CI) were calculated using logistic regression analysis, adjusted for different variants effected on lymphoma, including age, family history, viral hepatitis, and blood group. Secondly, we calculated odds ratios for the more frequent lymphoma subentities (with n>30 cases). To increase the statistical power, patients with these lymphoma subentities were compared separately with the entire control group (n=200) using logistic regression analysis.

Our analyses for the associations of family history of cancer used only data on the first-degree relatives, because of the low reliability of data on second-degree relatives. The presence of hematopoietic cancer in at least one first-degree relative was considered a positive family history of hematopoietic cancer. A family history of cancer was defined as having cancers other than hematopoietic cancer among first-degree relatives.

A 5-mL blood sample was collected from each consenting participant and sent to Shiraz Blood Transfusion Organization. Both, cases and controls underwent; a) ABO blood typing and b) Enzyme-linked immunosorbent assay (ELISA) to test serum samples for hepatitis B surface antigen (HBsAg), hepatitis C antibody (anti-HCV), and HIV antibody.

According to the WHO classification, lymphoid neoplasms were grouped in B cell lymphoma (lym-phoplasmatic lymphoma, splenic marginal zone lymphoma, marginal zone B cell lymphoma, follicular lymphoma, diffuse large cell lymphoma, other B cell lymphoma), T cell lymphoma (mycosis fun-goide/Sezary, other T cell), and Hodgkin’s lymphoma. Multiple myeloma was reported separately in this study, but belonged in the classification of B cell lymphoma according to the WHO classification. The group of B cell lymphoma was thus analyzed with excluding multiple myeloma in this study.

Analyses were conducted for ever farming and for farming as longest occupation. Multinomial logistic regression was used to estimate the odds ratios and the 95% confidence intervals to measure association between working in farming and lymphoma risk. Farmers were compared to people who had never held a farming job, and pesticide exposed farmers were compared to non-exposed people. Standard statistical procedures were carried out using SPSS software (Version 15.0, Chicago, IL, USA). T-test and Chi-Square test for differences between groups were used to compare cases and controls with respect to age, sex, educational status, smoking and family history of cancer. Two-sided *p* values were considered statistically significant at the 0.05 level.

A strictly confidential specimen evaluation was conducted. Ethical approval for the study was obtained from the local ethics committee. All subjects gave their written informed consent for HBV, HCV and HIV screening tests.

## Results

Of all eligible subjects, 200 controls and 200 cases were included in the study. The corresponding fre-quencies for next of kin were 8% for cases and 12% for controls. Of all subjects, 212 (53%) were male. The mean (standard deviation) age among cases and controls were 42.31 (17.37) and 43.48 (16.9) years, respectively. No statistically significant differences were observed between cases and controls with respect to age, sex, or smoking, but there was a statistically significant difference (p=0.001) for educational status. Each subtype was compared separately with controls by Chi-Square for educational status. There was no difference between B-non-Hodgkin’s lymphoma (p=0.075), T-non-Hodgkin’s lymphomas (0.459) and Hodgkin lymphoma (0.241) with controls. However, multiple myeloma's had lower education. In Table 1, detailed information was described comparing each subtype with the control group.

**Table 1:** Age and sex distribution of patients with lymphoid neoplasms and controls at Nemazee Hospital, Shiraz, 2007-2008.

**Diagnosis**	**Age (years)**		**Mean (SD)**	**Sex [No. (%)]** **M/F Ratio**
	**Min**	**Max**		
B-NHL^a^	16	75	40.17 (17.5)	50 (69.4)/22 (30.6)=2.3/1
T-NHL	17	72	35.27(17.1)	9 (60)/6 (40)=1.5/1
HL^b^	16	70	30.46 (14.9)	28 (52)/26 (48)=1.1/1
MM^c^	36	85	61.9 (13)	30 (64)/17 (36)=1.8/1
Control	17	83	43.48(16.9)	110 (55)/90 (45)=1.2/1

^a^ NHL=non-Hodgkin's lymphoma,

^b^ HL=Hodgkin's lymphoma,

^c^ MM=Multiple myeloma

Among 200 lymphoma patients, 54 suffered from Hodgkin lymphoma, 72 from B-non-Hodgkin’s lymphoma, 15 from T-non-Hodgkin’s lymphoma, and 46 from multiple myeloma. Thirteen cases of non-Hodgkin's lymphoma were not classified. The distribution of malignant lymphomas is shown in Table 2. Most of the non Hodgkin's lymphoma cases in our population were classified as B cell lymphoma (n=72.72%) with the most common subtype being diffuse large B-cell lymphoma.

**Table 2:** Distribution of WHO-defined histologic types of malignant lymphoma at Nemazee Hospital, Shiraz, 2007-2008.^a^

**Type of histology**	**No. of cases**	**Percentage**
Non-Hodgkin's lymphoma B-type		
Diffuse large B cell	34	47.2
MALToma	2	2.7
Lymphoblastic	8	11.2
Burkitt's	13	18.1
Follicular	5	7
Anaplastic large cell	1	1.4
Other B-NHL unclassified	9	12.4
Total	72	100, 36
Non-Hodgkin's lymphoma T-type	15	7.5
Non Hodgkin's lymphoma unclassified	13	6.5
Hodgkin's lymphoma		
Lymphocyte predominant	4	7.5
Lymphocyte depleted	1	1.9
Mixed cellularity	19	35.4
Nodular sclerosis	21	38.5
Unclassified	9	16.7
Total	54	100, 27
Multiple myeloma	46	23
Total	200	100

^a^ Student has no code in ISCO (International standard classification of occupations code).

Our analyses for the associations of family history of cancer used only data on the parents, brothers, sisters, and children (first-degree relatives). Because of the low reliability of data on second-degree relatives, risk of non-Hodgkin's lymphoma in positive family history cases of malignancy increased 4.4 times that was statistically significant (95% CI=1.46-13.3); however no association was seen in Hodgkin's lymphoma and multiple myeloma.

Cases and controls occupations code were recorded similar to International Standard Classification of Occupations code (ISCO-68) in Table 3. Most of females in cases and controls were housekeeper. The significantly increased risks can be seen for farmers (p=0.021). There were 93 subjects (23.25% of study population) who could be identified as farmer. As compared to all other subjects, farmers were more likely to be male (96% versus 56%) (p=0.014), and to be of a lower educational status (45% of farmers were illiterate) compared to all other subjects.

**Table 3:** Occupation of patients with lymphoid neoplasms and controls at Nemazee Hospital, Shiraz, 2007-2008.

**ISCO code**	**Occupation**	**Case**	**Control**
1-3	Teachers	7	10
1-4	Workers in religion	2	1
0-6/7	Medical, dental, and related workers	3	4
3-0/9	Clerical and related workers	13	20
4-0/9	Sales workers	19	15
5-2	Housekeeping	61	73
6-2	Farmers	57	36
7/8/9	Production and related workers	10	8
0-2/3	Architects, engineers and related technicians	2	1
Y	Members of the Armed Forces	5	3
	Student^a^	17	13
	Unemployed, unknown usual occupation	4	16

^a^ Student has no code in ISCO (International standard classification of occupations code).

Only 42.5% (85/200) of the malignant lymphoma patients were available for testing by serology for HBS Ag, HCV Ab and HIV Ab during the study period and 2 (2.35%) tested positive including 1 non-Hodgkin’s lymphomas (B-cell type, Diffuse large B cell), and 1 Hodgkin's lymphoma (Mixed cellularity) while all screened multiple myelomas were seronegative. In controls, 6 subjects were positive including 2 HBS Ag, 3 HCV Ab and 1 HIV Ab. Due to difficulties in control group for viral assay, information from 400 donors referred to Shiraz Blood Bank were considered because of low positive cases, t test was used to compare all positive cases and controls dispensing with subtypes (p=0.547) that was not statistically significant. Blood group O was the most common blood group in controls (42%); however, blood group AB was a rare blood group in both cases and controls. Although most cases of non-Hodgkin’s lymphomas had group B and O and in multiple myeloma, group A with 34.7% had first rank but they were not statistically significant (p=0.06, 0.71). 75.5% and 87% of cases and controls were RH positive (p=0.349).

Table 4 shows the chemical agents to which subjects were exposed specially at work. As is seen, pesticides had first place (OR=3.8, 95% CI=1.7-8.5). Hair dyes and cleaners had abundant use in both cases and controls and13 cases lived in areas near petroleum and gas refinery.

**Table 4:** Number of exposed cases and controls with odds ratio and 95% confidence interval

**Agent**	**Number of exposed** **Cases/Controls**	**OR (95% CI )**
Asbestos	3/1	
Cleaner	35/42	
Petroleum	13/9	
Glass wool	6/4	
Hair dye	32/40	
Pesticide	81/45	3.8 (1.7-8.5)

Pesticide exposure, duration of exposure and kind of pesticides were shown in Table 5. First, we examined the associations with potential exposure to pesticides at work or home. There was an increase in risk of non-Hodgkin’s lymphomas, Hodgkin’s lymphomas and multiple myeloma with the number of years worked on a farm (p=0.053). The OR associated with such farm work of ≥ 17 years was 2.12 (95% CI, 1.21–3.71).

**Table 5:** Risk of lymphoma by pesticide exposure in patients admitted in Nemazee Hospital, Shiraz, Iran, 2007-2008.

**Agent **	**NHL^e^**	**HL^f^**	**MM^g^**
Any pesticide (No)			
Yes [1]	52	13	28
No [2]	48	41	18
OR (95% CI)†	3.9 (2.2; 6.8)	1.04 (0.4; 2.6)	2.48 (1.16; 5.2)
Duration of exposure			
High^a^ [1]	34	8	12
Low^b^[2]	18	5	6
OR (95% CI)****	2.12 (1.2; 3.7)	-	1.74 (1.03; 2.1)
Kind of exposure	Case/Control	OR^c^	95% CI^d^
Herbicide	41/35	0.59	1.1; 3.6
Crop insecticide	28/9	3.8	1.7; 8.5
Fungicide	14/15	-	-
Home pesticides****	18/21	0.52	0.2-1.3

^a^ High exposure is defined as >median number of years for exposed subjects.

^b^ Low exposure is defined as <median number of years for exposed subjects.

^c^ Odds Ratio and confidence interval adjusted for age,sex,family history of cancer and smoking and they were compared 1 with 2 in cases and controls.

^d^ due to low cases in each subtypes all cases and controls compared in nominal logistic regression with controls.

^e^ NHL=non-Hodgkin's lymphoma

^f^ HL=Hodgkin's lymphoma

^g^ MM=Multiple myeloma

Of all malignant lymphoma patients, 58 were farmers (29 B-non-Hodgkin’s lymphomas, 1 T-non-Hodgkin’s lymphomas, 12 Hodgkin’s lymphomas and 16 multiple myeloma). Mean age of farmers was 48.2 and 46.4 years in cases and controls, respectively. Among all farmers, 97% of them reported the use of any pesticide. Furthermore, only 19.14% of the all cases who used pesticides could recall the product names; thus, evaluation of the chemical class of pesticides was not feasible. When lymphoma risk was evaluated for the different exposure categories that were stratified by group of farming, insecticide was the only exposure category that was significantly associated with lymphoma risk among farmers other than general population. The mean (SD) duration of exposure to pesticides was 12.3 (13.0) years. An increased risk was observed for all farmers exposed to pesticides for >17 years. The mean (SD) frequency of exposures was 7 (17) days/year. When types of crops handled by the study subjects were considered, the OR appeared to be higher for vegetables, grain, and other crops than for fruits and flowers, although none of them was statistically significant. Farmers also were asked about safety methods during their spraying. About 50% of farmers did not use any protective methods and less than 5% used more than 2 devices.

## Discussion

Non-Hodgkin’s T-type lymphomas were 7.5% in the present study and 9.3% in western countries.[4] Thus; there is a lower incidence of non-Hodgkin’s T-type lymphomas in Iran compared with the West. Considering the degree of malignancy, the majority (77.9%) of B-type lymphomas of the present study are high-grade lymphomas (Diffuse large B cell, Lymphoblastic, Burkitt's, anaplastic large cell lym-phoma); however, the majority (54.5%) of lymphomas were of the low-grade type in western countries.[4] Follicular lymphoma with 30% is the most common type of non-Hodgkin’s lymphomas in the United States4 but only 7% of non-Hodgkin’s lymphomas cases in this study had follicular lymphoma. One reason for this difference could be due to the lower mean age of the population in Iran compared to western countries.

In this study, some etiologic factors of lymphoid malignancies were evaluated including family history of cancer, smoking, HBV, HCV and HIV viruses and occupational exposures. Our results confirmed previously reported associations of non-Hodgkin’s lymphomas and a history of cancer among first-degree relatives.[8] Risk of non-Hodgkin’s lymphomas in positive family history cases of malignancy increased 4.4 times that was statistically significant (95% CI=1.46-13.3); however no association was noticed in Hodgkin’s lymphomas and multiple lymphoma. It is opposed to several studies that showed increased risk of Hodgkin’s lymphomas and multiple myeloma in patients with positive history of hematologic cancers in their families.[4] This difference may be due to low number of cases in this study. Smoking was not a risk factor, confirming one study[9] and contradicting others,[10] although certain subtypes of non-Hodgkin’s lymphomas may be associated with cigarette smoking.[9][10]

Although we did not find a statistically significant association with serologically confirmed HCV, HBV and HIV infection in non-Hodgkin’s lymphomas, Hodgkin’s lymphomas and multiple myeloma cases, our point estimate was consistent with those from other countries with low prevalence of these viruses.[11]

Recent population-based data from large incident case series in the United States[12] and a systematic review[11] support a positive association between HCV infection and non-Hodgkin lymphoma risk. Several studies showed a positive association of HBV[13] and HIV[14] in lymphoma.

### Occupation and Chemical Exposures

In the data presented, people who had ever been a farmer had an increased risk of lymphoma compared to all other occupations. The hypothesis that farming, agricultural practices[4][14][15][16][17] and pesticide exposure[7][16][17][18][19] are associated with non-Hodgkin lymphoma has been tested in a number of occupational studies. Not all of the studies confirm an association.[6]

Two etiological hypotheses have been advanced to account for the frequently observed association between lymphatic and hematopoietic cancers and agricultural occupations. Farmers may be more frequently exposed to agricultural chemicals, including phenoxy herbicides, chlorophenols and pesticides.[17][19][20][21] Second, livestock farmers may be exposed to oncogenic animal viruses, particularly bovine leukemia virus. Bovine leukemia virus is related to HTLV-1 and reported to produce erythroleukemia in chimpanzees, therefore speculation exists about an association between exposures to cattle, and particularly those with bovine lymphosarcoma, and human leukemia incidence.[22] However, one case-control study[23] negate any association. McDuffie et al. actually found a reduction in risk of non-Hodgkin lymphoma among Canadian male farmers who raised cattle, although farmers who had a large (13+ head) swine inventory or raised bison, elk, or ostriches had increased non-Hodgkin lymphoma risk.[24]

We found that persons with exposure to any pesticide had increased the risk of lymphoid neoplasms [OR=0.95, 95% CI=(1.864-5.998)]. Exposure to pesticides increased risk of non-Hodgkin lymphoma [OR=3.9, 95% CI=(2.2-6.8)], and multiple myeloma [OR=2.48, 95% CI=(1.16-5.2)]. Although risk of Hodgkin lymphoma increased with pesticide exposure, it was not statistically significant [OR=1.04, 95% CI= (0.41-2.66)]. Further analysis of pesticides showed that patients who had been exposed for long periods had an increased risk of lymphoma. This is consistent with findings from another study restricted to women who lived and worked on a farm where pesticides were used for more than 10 years.[24]

Less than 20% of the all cases who used pesticides could recall the product names; thus, evaluation by chemical class of pesticides was not feasible. So, risk of lymphoid malignancies was evaluated in 4 pesticide categories: Insecticide, herbicide, fungicide and home pesticides. It was seen that risk of lymphoma increased in insecticide exposures. Most of farmers in this study were in middle age and 45% of farmers were illiterate compared to all other subjects.

Pesticides are mostly used by farmers, but other people (for example housekeepers exposed to home pesticides) can also be exposed, which makes comparison between farming studies and exposure studies difficult. In this study, we consider both groups. Moreover, farmers experience multiple exposures. Another problem in comparing the current study to others is that the investigated disease differs between studies and/or that a different classification system may be used. Classification systems differ according to the types of lymphomas included. We analyzed all lymphoma together as well as B cell, T cell, and Hodgkin’s lymphoma separately. Most studies that investigate the cancer risk among farmers only evaluate the risk of non-Hodgkin lymphoma and not for subtype.[16][19] Kato et al.,[24] evaluated subtypes of non-Hodgkin lymphoma with use of pesticides. They found increased risks for different levels of exposure among women exposed to pesticides for both B and T cell lymphoma and for high grade lymphoma. Our data identified an increased risk of B-cell lymphoma. As in all retrospective case control studies, the exposure to agents could not be measured directly and information for this study was based on lifetime recall from personal interviews.

Farmers exposed to pesticides were found to be at an increased risk of lymphoma. The risk was clearly observed for herbicides, insecticides and fungicides. This risk was greatest when exposure to pesticides was more than17 years. Furthermore, subjects exposed to pesticides were at an increased risk of non-Hodgkin lymphoma and multiple lymphoma, but not Hodgkin lymphoma.
